# Editorial: Metalloproteins as sensors of gaseous small molecules—From bench to bed and beyond

**DOI:** 10.3389/fmolb.2023.1140392

**Published:** 2023-01-19

**Authors:** Eduardo H. S. Sousa, Shiliang Tian

**Affiliations:** ^1^ Bioinorganic group, Department of Organic and Inorganic Chemistry, Federal University of Ceará, Fortaleza, Brazil; ^2^ Department of Chemistry, Purdue University, West Lafayette, IN, United States

**Keywords:** metalloprotein, iron sulfur cluster, hemeprotein, nitric oxide signaling, carbon monoxide signaling, biological sensing, oxygen sensing

During the last decades, the biological signaling role of a variety of small gaseous molecules has been unveiled, which has included molecules such as NO, CO, O_2_, ethylene, CO_2_, H_2_, H_2_S, and others (Ganesh et al., 2020; Lopes et al., 2021). How they are sensed and wired to responsive systems are under intense investigation with cases found in all kingdoms of life. Among these molecules, NO, CO and O_2_ stand out, where new signaling systems are still being discovered. Indeed, two Nobel prizes were awarded to studies covering some of these systems, one in 1998 for NO as a signaling molecule (R. Furchgott, L. Ignarro and F. Murad (Smith, 1998)) and 2019 to the particular O_2_ sensing system found in mammals (W. Kaelin, P. Ratcliffe and G. Semenza (Burki, 2019)).

**FIGURE 1:**
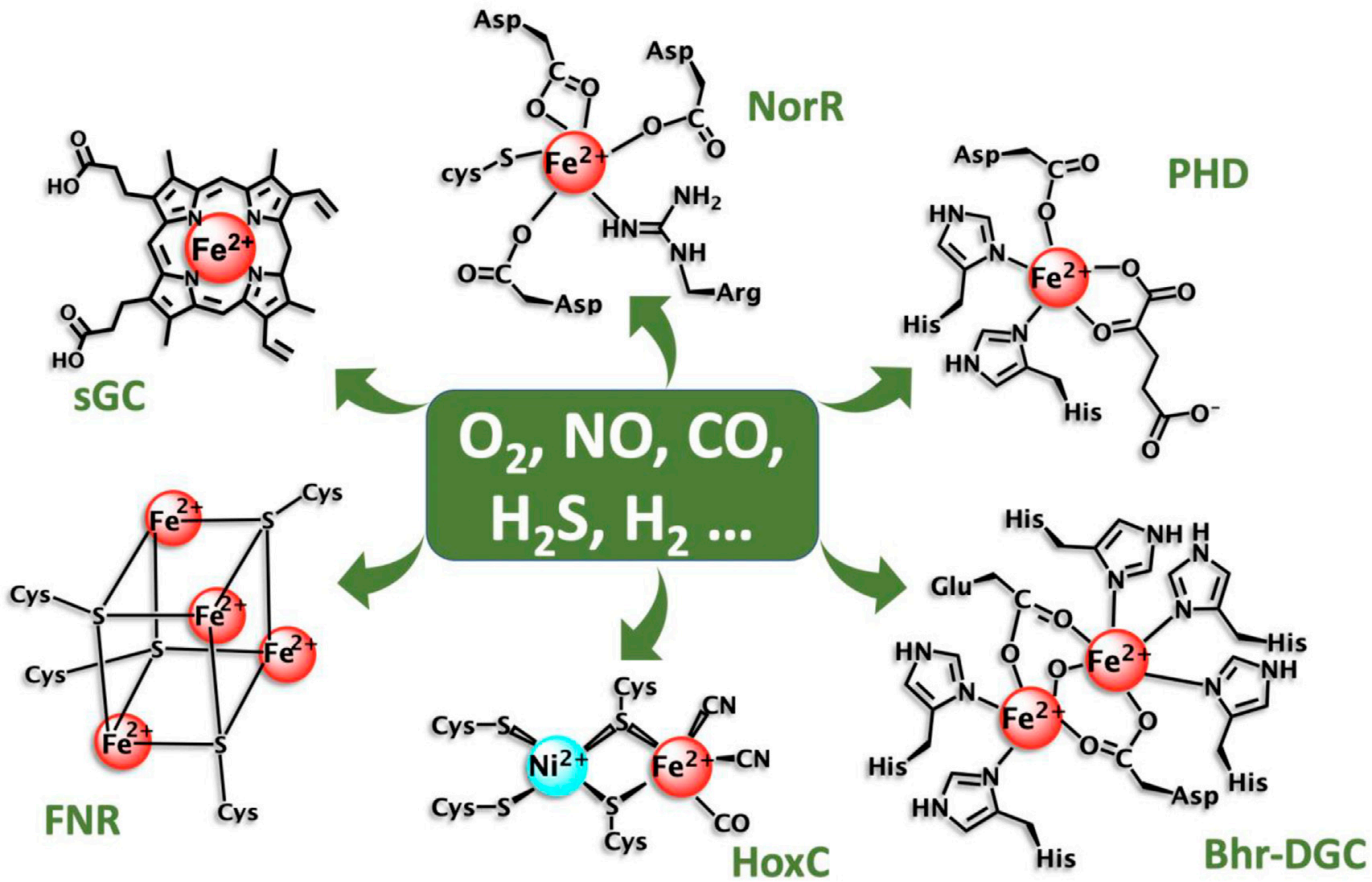
Some examples of metal sites employed by selected gas sensing metalloproteins.

To accomplish the sensing function of small molecules, Nature has mainly selected metalloproteins as key sensing units that are responsible for the interaction with the small molecules enabling a signal transduction event to take place. This process occurs through alterations of the protein conformation affecting responsive elements. These metalloproteins are quite variable using distinct metal ions (e.g., Fe^2+^ and Ni^2+^) directly bound to amino acid sidechains or through anchoring metal-containing cofactors (e.g., porphyrin) (Figure 1). Despite the remarkable number of gas-sensing metalloproteins that have been discovered, it is likely many more examples have yet been identified. Fundamental studies are still essential and many exciting applications are emerging from these systems (Lemon and Marletta, 2021; Gondim et al., 2022). The mammalian NO sensor, soluble guanylate cyclase (sGC), is one representative case, where two new drugs targeting this protein, Adempas and Verquvo, were approved by FDA for cardiovascular disorders in 2013 and 2021 respectively. Besides this, many other gas-sensing metalloproteins have been developed as biochemical or analytical tools, novel biocatalysts or transcriptional regulators of synthetic biology pathways (Gondim et al., 2022). This Research Topic has received exciting contribution highlighting this breadth.

In this Research Topic, Kitanishi presents a short review on the structural and functional role of hemerythrin-based O_2_ and redox sensors found in bacteria. These sensing metalloproteins are expanding during the last years, which are commonly associated to chemotaxis responses or enzymatic activities. The role of some of these sensors in c-di-GMP metabolism and biofilm regulation are discussed. Besides that, Kitanishi remarks further biotechnological interest in these proteins as well.

CO is toxic at high concentrations due to its inhibition of O_2_ transport by binding to the heme proteins. However, at low concentrations, CO is an important signaling molecule that mediates various signaling processes. The minireview by Vos et al. mainly focuses on the two unambiguously identified heme proteins: CO-activated transcriptional activator CooA and CO-responsive transcriptional regulator RcoM, where the binding of CO to the heme Fe(II) changes the heme coordination state and leads to the confirmational changes in the remote DNA-binding sites. In addition, the review also discusses other CO-responsive proteins such as human cystathionine β-synthase and nuclear receptor Rev-Erbβ.

In mammals, NO is a key biological messenger involved in several physiological and pathological processes. For instance, the endothelium of blood vessels utilize NO to signal the surrounding muscle to relax, resulting in vasodilation and increasing blood flow. Silva et al. comprehensively reviewed the current literature on the roles of NO in hypertension and the functions of the membrane metalloproteinase ADAM17 implicated with the NO production.

Fontenot et al.’s lab reported evidences on the reversible binding of NO to the unusual iron-sulfur cluster found in the MitoNEET protein. This mitochondrial membrane protein is involved in the regulation of energy metabolism, iron homeostasis and production of reactive oxygen species, which has been associated with some diseases. This study sheds further light on the role of NO in reversibly regulating electron transfer processes of this protein.

Soluble guanylate cyclase was the first heme-based sensor discovered, which is associated with multiple biological processes regulated by NO. This sensor has an unusual incapacity to bind O_2_, which may allow its efficient functionality as a NO sensor in mammals. Here, Wu et al. present a review exploring some reasons for this selectivity and the specific response to some gases such as NO, H_2_S and CO. The distinct mechanism of response to low and high levels of NO is also discussed enlightening the readers on this remarkable protein.

Altogether, this Research Topic combined some distinct and exciting sensing systems from bacteria to humans, where studies from core biochemistry to cell biology were explored. They have provided only a taste on the multiple flavors of this broad and vibrant field.
